# Army and Navy Dental Surgery

**Published:** 1899-11

**Authors:** B. H. Catching

**Affiliations:** Atlanta, Ga.


### ARMY AND NAVY DENTAL SURGERY.

BY B. H. CATCHING, D.D.S., ATLANTA, GA.

It does seem that we are on the road to having dental surgeons in the army and navy. The end of the road is not in sight yet, but, judging by the signs, it is not far off. It is through the National Dental Association the profession should speak, and the action of that body on this question is anxiously awaited.

We need not argue the necessity for such a department, for there is not a single argument against it, save expense to the government, and that argument will not stand by the side of humanity and duty.

England is awake on this line; and before we know it, she will be in advance of us. She has not the politicians to fight or to win that we have.

I quote as follows from the *British Journal of Dental Science:*

"Soldiers' Teeth.—The fiat has gone forth from the War Office that in the London and Manchester recruiting districts the examining medical officer shall have the power of obtaining dental services for an otherwise acceptable recruit. There seems to be great joy among the recruiting sergeants, among whom the regulation forms an absorbing topic. Several agreed that it was more than time something of the sort was done, as many fine, big fellows were rejected daily on the score of having 'more than five unsound teeth.' In very rare instances 'pictures of men' had been admitted with artificial teeth. It was a *sine qua non* that the soldier should be able to masticate his meat. To escort a recruit to the dentist's would be a new experience; though there was a division of opinion as to whether the new regulation would attract or scare. At the Admiralty Head-quarters, in Spring Gardens, the head clerk explained that the new order did not extend to the navy. They declined hundreds of men in the year on account of their dental deficiencies, being still more particular than in the army. The marine recruiting sergeants had more to say, feeling the teeth question very keenly: 'Before a man can join the royal navy he must have four sound opposing molars, and men must gape before we think of taking them on.' It was, they complained, an every-day occurrence for fellows to walk straight from them to St. George's barracks and join the army."

The obtaining of dental services at the recruiting stations will lead on to a permanent demand for his services. I have read with interest Dr. Schamberg's article in your June issue, and fully agree with him that only those having the M.D. degree with the D.D.S. degree, should be eligible.

Lately we have seen where the War Office had to reduce the requirements at recruiting stations. I do not know where the reductions were made, but it shows that if we are to maintain a larger standing army and maintain high requirements for enlistment, sooner or later the office of the dental surgeon will be in demand. I have seen already, in anticipation of this act, young men seeking influence for the position who are not competent to fill it.

I find that a common sentiment in favor of this movement is fast forming. The necessity for services of the dental surgeon was made manifest in the Cuban campaign; it was brought close home to many who are now active in public life, whose counsel will aid the movement.

The forming of a new army and navy opens this question to the eyes of many in authority who have been blind to its necessity under the old standing army regulations.

Keep this question, it is an important one.

